# On the prospect of an experimental account of argumentation. Commentary: Toward an experimental account of argumentation: the case of the slippery slope and the ad hominem arguments

**DOI:** 10.3389/fpsyg.2016.00299

**Published:** 2016-03-02

**Authors:** John Ian K. Boongaling

**Affiliations:** Division of Philosophy, Department of Humanities, University of the Philippines Los BañosLos Baños, Philippines

**Keywords:** argumentation theory, epistemic aspects of argumentation, logic, persuasion, reasoning

Lillo-Unglaube et al. (2014) argue for the employment of more descriptive and experimental accounts on the psychology of argumentation. By utilizing experimental studies concerning the slippery slope and *ad hominem* arguments using Bayesian (Hahn and Oaksford, 2007) and pragma-dialectic frameworks (Van Eemeren and Grootendorst, 2004), they seek to show how psychology can bring “the cognitive and normative accounts of argumentation closer” (Lillo-Unglaube et al., 2014, p. 5) for an integrated area of research on the psychology of argumentation.

Argumentation is a *complex* human activity. Its complexity stems from the fact that cognitive agents in various situations perform multiple tasks in real-time to assess an argument presented to them. These tasks involve the *quantity, quality*, and *relevance* of the statements that make up the argument. To illustrate, suppose cognitive agent A is presented with an argument for assessment. The quantity of statements included in the premise set has repercussions on what A can correctly infer (e.g., an argument with a single statement for a premise greatly reduces the number of inferential moves that A can make). It is also important for A to consider the quality of the statements included in the premise set (i.e., whether or not they are true). Finally, A must also consider the relevance of the statements included in the premise set to the conclusion of the argument (i.e., the premise set must be connected to the conclusion). As an additional complication, most of the time, cognitive agents are characterized by a condition of *uncertainty*. For example, all the relevant premises are oftentimes unavailable to cognitive agents, they are not always “in a position to know” (Sosa, 1995, p. 28), for instance, whether or not the premises of an argument are true.

Lillo-Unglaube et al.'s paper seeks to explain the “rhetorical effectiveness” of some of the informal fallacies. For Lillo-Unglaube et al., when cognitive agents are confronted with the slippery slope and *ad hominem* arguments, they evaluate “the persuasiveness of the slippery slope argument or the degree of unreasonableness of the *ad hominem* argument” (2014, p. 5).

It needs mentioning that Lillo-Unglaube et al.'s work benefited immensely from the works of Hahn and Oaksford (2007). For Hahn and Oaksford, the Bayesian approach helps explain how some informal fallacies can be *reasonable* inferential moves in *some* argumentation situations. Viewed in its proper context (i.e., that the inference involved in the slippery slope and *ad hominem* arguments is *inductive*), one can safely say that Hahn and Oaksford's analysis is concerned with *argument strength* which can be understood in terms of *degrees* unlike *logical validity*. This creates a potential problem for Lillo-Unglaube et al.'s proposal. Suppose cognitive agent A is persuading B that C will cheat in the upcoming final examinations on the basis that C cheated in their final examinations in the previous terms. To be sure, such an argument is persuasive or reasonable to hold. However, from the point of view of *traditional* normative approaches, there is a straightforward answer as to why A's argument is *defective*: its conclusion is not a *logical consequence* of the premise set. From the point of view of the experimental account by Lillo-Unglaube et al., however, the persuasiveness and the degree of reasonableness of A's conclusion would depend on the prior probabilities that we attach to the premise set. Mindful of the fact that these two approaches are different in the sense that the *nature* of the inferences involved are different (i.e., logical validity applies to deductive whereas argument strength applies to inductive arguments), one may ask as to how Lillo-Unglaube et al. can hope to bring these approaches closer together. If this is correct, then it is also unclear as to how Lillo-Unglaube et al. can maintain that the “psychology of argumentation provides an integrative scientific perspective unlike normative or aprioristic approaches” (2014, p. 5).

An option that is available for Lillo-Unglaube et al.'s proposal is to explain rhetorical effectiveness (e.g., persuasiveness, degree of unreasonableness) in relation to *truth*—a central *epistemic* concept that cognitive agents aim at (Corner and Hahn, 2013). This is in line with Hahn and Oaksford's (2006a) view that the Bayesian approach has considerable potential for advancing epistemic approaches to argumentation and evident in the very manner by which the Bayesian approach via probability theory provides explanations for the quality of arguments that cognitive agents encounter in real life (Hahn and Oaksford, 2006b; Corner et al., 2011; Hahn and Hornikx, 2015). As is well-known, the Bayesian approach interprets probabilities (which are measured in numbers from 0 to 1 where the probability of a necessary truth is 1) as “subjective degrees of belief” (Hahn and Oaksford, 2007, p. 707). Another advantage is that truth is a central notion to *inference* whether deductively or inductively. For example, it is truth that is supposedly *preserved* in deductively valid arguments (i.e., *if* the premise set is true, then the conclusion *must* be true). In addition, it is the truth of the conclusion in relation to the premise set that may vary in *strength* (e.g., weak, strong) in the case of inductive arguments. Finally, it can accommodate the lone reasoner by highlighting the idea that argumentation has an *intrapersonal* aspect evident in *belief formation* as well as *belief change* (Hahn and Hornikx, 2015). For instance, in deciding *what to believe in*, a cognitive agent interprets all the available evidences before her and then makes an inference. In the process, the cognitive agent may form beliefs that are *epistemically responsible*. A belief is epistemically responsible “if an agent holds those beliefs on the basis of good reasons, with the aim of attaining her epistemic goal. Both the reasons and the goal are evidently part of the agent's cognitive perspective” (Crumley, 2009, pp. 162–163).

In the end, if the goal is to have an integrated area of research on the psychology of argumentation, Lillo-Unglaube et al.'s proposal would benefit by including the notion of truth in explaining the rhetorical effectiveness of the slippery slope and *ad hominem* arguments in some contexts.

## Author contributions

The author confirms being the sole contributor of this work and approved it for publication.

### Conflict of interest statement

The author declares that the research was conducted in the absence of any commercial or financial relationships that could be construed as a potential conflict of interest.
